# Optical Coherence Tomography Angiography Assessment of the Optic Nerve Head in Patients Hospitalized Due to COVID-19 Bilateral Pneumonia

**DOI:** 10.3390/medicina60030502

**Published:** 2024-03-19

**Authors:** Magdalena Kal, Michał Brzdęk, Dorota Zarębska-Michaluk, Antonio Pinna, Jerzy Mackiewicz, Dominik Odrobina, Mateusz Winiarczyk, Izabella Karska-Basta

**Affiliations:** 1 Collegium Medicum, Jan Kochanowski University, 25-317 Kielce, Poland; kalmagda@gmail.com (M.K.); michal.brzdek@gmail.com (M.B.); dorota1010@tlen.pl (D.Z.-M.); 2 Ophthalmic Clinic of the Voivodeship Hospital in Kielce, 25-736 Kielce, Poland; magdale_5@hotmail.com; 3 Department of Infectious Disease, Provincial Hospital in Kielce, 25-317 Kielce, Poland; 4 Ophthalmology Unit, Department of Medicine, Surgery, and Pharmacy, University of Sassari, 07100 Sassari, Italy; apinna@uniss.it; 5 Department of Vitreoretinal Surgery, Medical University of Lublin, 20-079 Lublin, Poland; jerzymackiewicz@umlub.pl (J.M.); winiarm86@gmail.com (M.W.); 6 Institute of Medical Science, Jan Kochanowski University, 25-317 Kielce, Poland; 7 Department of Ophthalmology, Faculty of Medicine, Jagiellonian University Medical College, 31-501 Krakow, Poland; 8 Clinic of Ophthalmology and Ocular Oncology, University Hospital, 31-501 Krakow, Poland

**Keywords:** COVID-19, radial peripapillary capillary network, optical coherence tomography angiography, optic nerve head

## Abstract

*Background and objectives*: We aimed to investigate changes in the radial peripapillary capillary (RPC) network using optical coherence tomography angiography (OCTA) in patients who recovered from coronavirus disease 2019 (COVID-19). *Materials and Methods*: This was a prospective study of patients hospitalized due to COVID-19 bilateral pneumonia between March and May 2021. The control group included healthy individuals matched for age and sex. Two months after discharge, the patients underwent ophthalmological examination, including optical coherence tomography (OCT) imaging. The RPC network and retinal nerve fiber layer (RNFL) of the optic disc (RNFL optic disc) were automatically evaluated and compared between the study groups. Additionally, the RPC parameters were compared between the men and women in the COVID-19 group, and correlations between the RPC and RNFL optic disc parameters were assessed. *Results*: A total of 63 patients (120 eyes) with bilateral pneumonia caused by severe acute respiratory syndrome coronavirus 2 infection were examined. No ophthalmic symptoms were reported by the patients. No significant differences were observed in the RPC parameters between the patients from the COVID-19 group and the 43 healthy controls. Moreover, the RPC parameters did not differ between the men and women in the COVID-19 group. A positive correlation was found between the RPC and RNFL optic disc parameters in the COVID-19 patients (*p* < 0.001). *Conclusions*: No changes in the RPC network were observed among the patients with COVID-19 bilateral pneumonia in the early period after hospital discharge. However, a longer follow-up is needed to monitor COVID-19–related changes in the microvasculature of the optic nerve head.

## 1. Introduction

Coronavirus disease 2019 (COVID-19) is a disease caused by severe acute respiratory syndrome coronavirus 2 (SARS-CoV-2). It was first identified in late 2019 and was declared a global pandemic by the World Health Organization in March 2020 [1]. While COVID-19 primarily targets the respiratory system, mounting evidence from diverse studies suggests that it can invade other body systems, including the central nervous system (CNS) and the peripheral nervous system. This results in a spectrum of neurological disorders, such as encephalitis, encephalopathy, Guillain-Barre syndrome, meningitis, and musculoskeletal symptoms [1]. However, despite these neurological complications, the neuroinvasive mechanism of SARS-CoV-2 remains unknown [1].

SARS-CoV-2 binds to the angiotensin-converting enzyme 2 (ACE2) receptors and transmembrane serine protease 2 (TMPRSS2), enabling the infection of the retina and the CNS [1,2], with viral RNA detected even in the cerebrospinal fluid [3,4]. This leads to circulatory disorders and thrombotic microangiopathy characterized by vascular endothelial damage, coagulopathy, and endothelial inflammation, as confirmed via the brain biopsies of patients who died due to COVID-19 [5,6,7]. While most affected adults present with respiratory symptoms, emerging data highlight the neurological manifestations of COVID-19, with CNS symptoms (mainly headaches) reported in 10% to 20% of patients [8]. Additionally, studies have documented various neurological complications, such as seizures, cerebrovascular events, encephalitis, and neuromuscular disorders, suggesting either direct CNS invasion or neurological consequences of viral infection [9,10].

Optical coherence tomography (OCT) is a modern noninvasive imaging technique that enables the qualitative and quantitative assessment of the retina, choroid, and optic nerve. OCT angiography (OCTA) is a recent extension of OCT technology that allows the quantitative evaluation of macular vessel density (VD) and the radial perivascular capillary (RPC) network located between the outer border of the retinal nerve fiber layer (RNFL) and the inner limiting membrane [11]. Recent studies based on OCT findings in children recovered from COVID-19 and retinal biopsies from deceased COVID-19 patients suggested potential alterations in the RNFL and ganglion cell layer [2,9]. In our previous studies, we described microvascular changes in the retina and choroid assessed via OCTA in patients with COVID-19 [12,13].

Both hypoxia and excessive proinflammatory cytokine production are implicated in retinal ganglion cell damage. Hypoxia induces glutamate release and elevates free radical production, thus significantly contributing to retinal ganglion cell death. These mechanisms were described in other hypoxia-inducing conditions, such as retinal artery or central retinal vein occlusion, glaucoma, diabetes, chronic obstructive pulmonary disease, carotid artery stenosis, Takayasu arteritis, hyperosmolar viscosity syndrome, and post-traumatic conditions (e.g., Purtscher retinopathy) [14,15,16].

The retina, optic nerve, and CNS have high oxygen demand, which renders them particularly susceptible to hypoxic insults. Such insults may culminate in the demise of retinal ganglion cells and their axons, which form the optic nerve [17]. Therefore, it is crucial to understand the consequences of hypoxia on the microvasculature in patients with severe COVID-19. The present study aimed to investigate changes in the RPC network assessed via OCTA imaging among patients hospitalized due to COVID-19 bilateral pneumonia. The findings from this study may have important implications because (1) the RNFL and retinal ganglion cells are involved in transmitting light stimuli to central visual centers, and (2) retinal ganglion cells form the optic nerve disc and connect the retina to the CNS. Considering the transmission of other viruses (e.g., herpes simplex virus type 1) via the ascending pathway through the optic nerve to the CNS, it is important to explore the potential involvement of this pathway in SARS-CoV-2 transmission to the CNS [18].

## 2. Materials and Methods

### 2.1. Subjects

This was a prospective study that included 63 patients (120 eyes) with COVID-19 bilateral pneumonia who were admitted to the Department of Infectious Diseases of the Regional Hospital in Kielce, Poland, between March and May 2021. The disease was caused by the B.1.1.7 variant of SARS-CoV-2. The infection was confirmed via a positive polymerase chain reaction (PCR) or rapid cassette antigen test (Abbott, IL, USA), and pneumonia was confirmed via chest-computed tomography scans displaying typical lesions.

The control group included 43 healthy sex and age-matched patients who presented to the ophthalmology clinic for a routine eye examination. The inclusion criteria for the control group were as follows: an age from 30 to 70 years, negative laboratory test results for SARS-CoV-2 infection (PCR from a nasopharyngeal swab), the absence of current or past COVID-19 symptoms, no close contact with patients with COVID-19 within the 14 days before the examination, and an absence of concomitant eye diseases.

The exclusion criteria for the entire study population included systemic conditions, such as diabetes mellitus, stroke, myocardial infarction, and autoimmune diseases. Additional ocular exclusion criteria were as follows: myopia exceeding −3 diopters, hyperopia greater than +3 diopters, central and peripheral retinal disorders, optic nerve disorders, a history of intraocular surgery, uveitis, ocular injury, and opaque media affecting the quality of the OCT scan.

Initially, 94 patients (188 eyes) were examined 2 months after hospital discharge. Patients with age-related macular degeneration (*n* = 10), diabetes mellitus (*n* = 13), glaucoma (*n* = 2), myopia exceeding –3 diopters (*n* = 3), hyperopia greater than +3 diopters (*n* = 1), and previous cataract surgery (*n* = 2) were excluded from the study. From the remaining group of 63 patients after COVID-19, we excluded 2 eyes with hyperopia exceeding +3 diopters, 1 eye with myopia greater than –3 diopters, 2 eyes after ocular injury, and 1 eye treated for uveitis.

### 2.2. Optical Coherence Tomography Angiography

**Figure 1:**
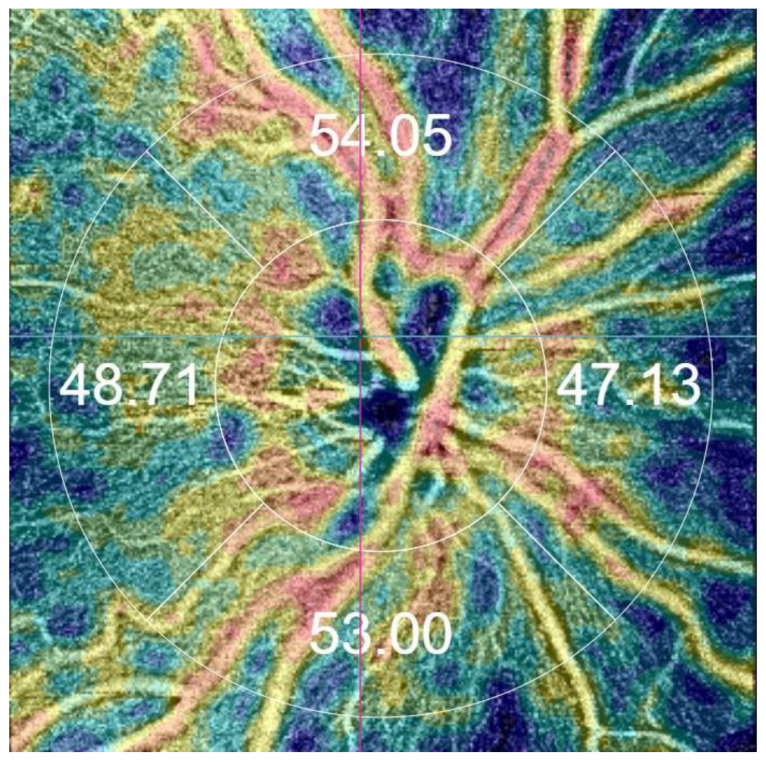
The image of the radial peripapillary capillary (RPC) network in the right eye was obtained via optical coherence tomography angiography (OCTA) automatically using the grid proposed in the early treatment diabetic retinopathy study (ETDRS). The automatic map of the capillary density of the optic nerve head was divided into four areas: the superior area (RPC S = 54.05%), the temporal area (RPC T = 48.71%), the inferior area (RPC I = 53.00%), and the nasal area (RPC N = 47.13%). The peripapillary area was scanned using a scan of 4.5 × 4.5 mm centered on the optic disc. The OCTA image was obtained with SS-OCT Triton (author’s archive).

Swept-source OCT (SS-OCT) was performed using DRI-OCT Triton (Topcon Inc., Tokyo, Japan). The peripapillary region was scanned using a scan of 4.5 × 4.5 mm centered on the optic disc. The parameters of the RNFL of the optic disc (RNFL optic disc) in the peripapillary area were assessed via OCT using a three-dimensional disc scan of 6 × 6 mm centered on the optic disc. The RPC network was automatically separated with the OCT instrument software (IMAGEnet, v. 61.34.19388, Topcon), extending from the internal limiting membrane to the posterior boundary of the RNFL. The capillary density in the RPC network was quantitatively assessed as the percentage of the measurement area occupied by the vessels. The software automatically fitted the inner circle to the optic disc, as per the early treatment diabetic retinopathy study (ETDRS) chart. The four areas of the RPC (i.e., superior, nasal, temporal, and inferior) dividing the center on the optic disc were automatically displayed. The mean RPC value was calculated as the average of the values obtained for the above four RPC areas (Figure 1). Scans with an image quality of at least 65% were considered eligible for analysis. The term “image quality” refers to the clarity, resolution, and absence of artifacts in the obtained scans. In this context, it encompasses the ability to discern the relevant anatomical structures and details within the images. It should be noted that SS-OCT, characterized by a scanning speed of 100 kHz and a wavelength of 1.050 nm, facilitates the acquisition of clear and detailed images, capturing even the deepest layers of the eye within a short acquisition time [19]. In SS-OCT, the axial resolution is 2.6 µm, the lateral resolution is 14 µm, and the scan width is 12 mm. Infrared light is less scattered, allowing better visualization of the posterior vitreous cortex and its structure. Considering the above properties, SS-OCT enables the simultaneous acquisition of high-quality images of the vitreous body, retina, choroid, and sclera [20].

### 2.3. Statistical Analysis

The demographic and imaging data were assessed using frequency and descriptive statistics. The quantitative variables were presented as a mean with a standard error of the mean (SEM) and a median with an interquartile range. The differences in the quantitative variables were compared between the study groups using the Mann-Whitney test and Student *t*-test. The clinical variables and RPC parameters were compared using the Student *t*-test. The categorical variables were presented as counts and percentages, and the comparisons between the groups were performed with the chi-square test. Spearman rank correlation coefficients were calculated to evaluate the significance, direction, and strength of the relationship between the RCP and RNFL optic disc parameters. J. Guilford’s classification was applied to interpret the correlation values. Counts and percentages were calculated for the qualitative variables, such as hypertension, dyslipidemia, and oxygen therapy. The effect size was determined using Cohen’s d statistic to assess the magnitude of the observed differences between the study groups. The level of significance was set at *p* < 0.05. The statistical analysis was performed using the Polish version of the STATISTICA 13.3 statistical package (STATSOFT, Kraków, Poland).

### 2.4. Ethical Considerations

The study was approved by the Bioethics Committee of the Collegium Medicum of Jan Kochanowski University in Kielce, Poland (study code 54, approved on 1 July 2021). The patients provided written consent for their ophthalmologic examination.

## 3. Results

The study population included 63 patients (120 eyes) who recovered from COVID-19 (mean [SEM] age, 51.33 [1.45] years) and 43 healthy controls (83 eyes; mean [SEM] age, 47.76 [1.38] years). There were no significant differences between the groups in age (*p* = 0.087) or sex (*p* = 0.515). During the ophthalmological examination, no ocular symptoms were reported by the patients in the COVID-19 group. The median LogMar visual acuity was 0.0 (0.0), and the mean LogMar reading vision was 0.3 (0.0), both in the COVID-19 and control groups. The mean (SEM) intraocular pressure was 16.16 (0.24) mmHg in the COVID-19 group and 16.36 (0.34) mmHg in the control group (*p* = 0.623). In the COVID-19 group, there were 43 men with a mean (SEM) age of 55.1 years (11.0) and 20 women with a mean (SEM) age of 49.7 (12.07) years. The mean (SEM) body mass index was 29.14 (3.54) kg/m^2^ for men and 26.8 (4.71) kg/m^2^ for women. Hypertension was reported in 19 patients (30.16%; 15 men and 4 women) during hospitalization, and dyslipidemia was present in 3 patients (4.76%; all male). Oxygen therapy was required in 22 patients (34.92%; 16 men and 6 women). The demographic, ocular, and systemic characteristics of the study population are summarized in Table 1.

No significant differences were observed between the COVID-19 and control groups in optic nerve head (ONH) parameters, such as the cup-disc ratio and rim area. The comparison of the ONH parameters between the study groups is presented in Table 2.

The comparison of the RPC parameters between the COVID-19 and control groups revealed no significant differences in the superior (*p* = 0.432), nasal (*p* = 0.754), inferior (*p* = 0.943), and temporal (*p* = 0.559) areas or in the mean RPC value (*p* = 0.669) (Table 3). The size effect was lower than 0.15, indicating a small size of effect.

The comparison of the RPC parameters between the men and women from the COVID-19 group showed no significant differences in the superior (*p* = 0.107), nasal (*p* = 0.303), inferior (*p* = 0.709), and temporal (*p* = 0.266) areas or in the mean RPC value (*p* = 0.559) (Table 4). The effect size was lower than 0.15, indicating a small size of effect.

In the COVID-19 group, the correlation analysis revealed positive correlations between the RPC and RNFL optic disc parameters in the superior (*p* < 0.001), nasal (*p* < 0.001), inferior (*p* < 0.001), and temporal (*p* < 0.001) areas. Additionally, correlations were observed between the mean RPC and mean RNFL optic disc values (*p* < 0.001) (Table 5).

In the control group, the correlation analysis also revealed positive correlations between the RPC and RNFL optic disc parameters across the superior (*p* < 0.001), nasal (*p* < 0.001), inferior (*p* < 0.001), and temporal (*p* < 0.001) areas. Moreover, a positive correlation was observed between the mean RPC and mean RNFL optic disc values (*p* < 0.001) (Table 6).

## 4. Discussion

In this study, we investigated changes in the RPC network among patients with a history of hospitalization due to COVID-19 bilateral pneumonia caused by SARS-CoV-2. Using OCT imaging, we aimed to identify any alterations in the RPC parameters caused by the disease.

OCT is a noninvasive tool for evaluating the histological anatomy of the retina, choroid, and optic nerve in various diseases. It facilitates the diagnosis and monitoring of patients with diabetes mellitus, primary open-angle glaucoma, and central serous chorioretinopathy [21]. Due to the variety of OCT systems currently available on the market, including spectral-domain (SD-OCT) and SS-OCT technologies, it is challenging to reliably compare the results among different studies. Both the SD-OCT and SS-OCT systems enable the visualization of the RPC network.

OCTA, which is a recent advancement in OCT technology, offers a novel approach to visualize the blood flow in retinal vessels and choriocapillaries without the use of contrast. This technique allows the assessment of retinal structure and microcirculation. The OCTA technology is based on the imaging of contrast between moving blood molecules and a still background. It is a static method as opposed to dynamic methods, such as fluorescein angiography. In the dynamic method, the contrast flow varies according to the duration of the examination, while in the static method, the flow in the vessels is detected at any time, and the image obtained is constant. In addition, OCTA allows the visualization of vessels in different layers of the retina: the superficial choroid plexus and the deep choroid plexus, at the level of the outer retinal layers and in the choriocapillaries. The scanning speed in OCTA is 70,000 scans A/s. However, OCTA scans can show only the pathological vascular flow and not all elements of the lesion or leakage [22]. Additionally, SS-OCTA, with a high A-line frequency of 100 kHz and deep signal penetration to the sclera, allows the segmentation and layer-by-layer analysis of the microvascular plexus of the retina and optic nerve disc. Moreover, the technology allows the creation of automatic thickness and volume maps of the retinal and choroidal layers. It is also possible to obtain OCT scans of much better quality in patients with reduced transparency of the optical centers, including cataracts and unfavorable refractive errors. Due to the large maximum width of the scans, SS-OCT cameras have the ability to simultaneously visualize the macular and disc area of the optic nerve. Finally, high transmittance and low light scatter mean that it is possible to obtain high-quality, artifact-free cross-sections of the anterior segment of the eye [20].

Macular and optic disc diseases can also be diagnosed with SD-OCT, which provides information about the internal structure of an object by measuring the signal of interfering waves recorded as the function of frequency. SD-OCT is 10 to 100-fold faster than standard OCT; thus, it can provide more A-scans. The higher number of A-scans increases the sensitivity and quality of the two-dimensional images obtained. In the commercially available version, the axial resolution is 3–6 µm, and the transverse resolution is 12–18 µm. The improved resolution of SD-OCT enhances the ability to visualize the retinal layers, as compared with standard OCT. In addition, optical zoom means that it is possible to magnify the image 1–3 times. SD-OCT enables three-dimensional imaging, with individual scans separated from each other by 0.14 mm. Scanning speeds range from 27,000 to 68,000 scans A/s, which effectively reduces motion artifacts. The width of the scans ranges from 6 mm to 9 mm. It is also possible to obtain retinal thickness maps, nerve fiber thickness maps, retinal pigment epithelium deformation maps, and thickness maps of the photoreceptor layer and retinal pigment epithelium. However, an SD-OCT camera does not support the quantification of VD in the macula and RPC network [23,24,25,26].

In our study, we did not identify differences in the RPC parameters between the COVID-19 patients and the healthy controls. Abrishami et al. [27] compared the ONH vascularization parameters between 25 patients with COVID-19 (mean [SD] age, 44.31 [1.93] years) and 22 healthy patients (mean [SD] age, 49.94 [2.22] years). The OCTA scans were obtained using the AngioVue device. The mean whole-image small vessel (SV) VD in patients with COVID-19 did not differ from that in the healthy group (*p* = 0.308). However, the authors noted a decrease in RPC VD in all vessels and SV, which was significant in the whole peripapillary SV VD, peripapillary inferior nasal SV VD, peripapillary inferior temporal SV VD, peripapillary nasal SV VD, and grid-based all vessel VD inferior sector (*p* < 0.05). A higher SV VD was observed inside the disc in the COVID-19 group compared with the control group (*p* = 0.021) [11]. In their previous work, the same authors also found a thickening of the RNFL optic disc in patients who recovered from COVID-19 [27]. The increase in SV VD inside the optic disc may be associated with edema and the hyperemia of the ONH, indicating possible optic nerve involvement in SARS-CoV-2 infection.

In our study, patients in the COVID-19 group did not show edema or congestion of the optic disc. This contrasts with the results of our previous study, in which a significantly thicker RNFL optic disc in the superior (*p* = 0.021) and inferior (*p* = 0.010) areas was present in a similar population [13]. Burgos-Blasco et al. [9] suggested that neuronal cell edema secondary to neuroinflammatory injury might contribute to increased RNFL optic disc thickness. This mechanism is similar to that of edema in the CNS.

Another study compared the RPC network between post-COVID-19 patients and a healthy group using AngioPlex OCTA. The study involved 90 patients (mean [SD] age, 55.48 [8.93] years), assessed at 4 and 12 months after confirmation of SARS-CoV-2 infection via PCR, and 29 healthy controls (mean [SD] age, 52.83 [8.49] years). The results indicated an increased VD in the temporal sector of the ONH in the COVID-19 group (*p* < 0.0001). However, no difference in the VD of the ONH was observed between the two follow-up visits at 4 and 12 weeks. Nevertheless, the study had several limitations. Patients with COVID-19 were not assessed during the acute phase of the disease during hospitalization, and the healthy control group was relatively small. In addition, the study did not include patients with severe illness, some of whom may have died of the disease [28].

Savastano et al. [29] reported a lower VD of the RPC plexus in 80 patients following SARS-CoV-2 infection (mean [SD] age, 52.9 [13.5] years) compared with 30 controls (mean [SD] age, 48.5 [13.4] years) (*p* < 0.04). An OCTA examination with the Spectral Domain Zeiss Cirrus 5000-HD-OCT Angioplex was conducted one month after hospital discharge. These findings appear to contradict those reported in the literature. The discrepancies may be due to the small control group, the relatively young age of the study population, and the mild course of the disease in COVID-19 patients who did not require intensive care treatment [29].

In our study, no differences were found in the RPC parameters between the men and women. To the best of our knowledge, this is the first study to assess the capillary density of ONH parameters according to sex. Therefore, a comparison of our results with other studies is not possible at present. In our previous study, we observed differences in the macular parameters between men and women as assessed via OCTA. Specifically, we found that the foveal VD of the superficial capillary plexus and the temporal VD of the deep capillary plexus were significantly reduced in women compared with men (*p* = 0.008; *p* = 0.018, respectively). Additionally, the foveal avascular zone in the superficial capillary plexus was significantly enlarged in women (*p* = 0.008) [13].

Previous research showed greater central retinal ischemia in women compared with men with SARS-CoV-2 infection [13]. Some investigators suggested that women may recover more slowly from COVID-19 than men [30,31]. Further studies are needed to identify the sex-related factors influencing the differences in the central retinal and ONH parameters on OCTA. Perhaps differences in the vascularization of the central retina and ONH should be considered. First, the surface of the optic nerve disc is supplied by the capillaries of the central retinal artery [32]. Second, the retinal ganglion cell axons in the peripapillary layer of nerve fibers are vascularized by a separate network of capillaries originating from the adjacent arterioles. These vessels of the RPC plexus run parallel to the nerve fibers [33]. Third, the short posterior ciliary arteries supply blood to the anterior lamina cribrosa, while the Zinn-Haller arterial circle and the peripapillary choroidal vessels contribute to the blood supply to the anterior lamina cribrosa [34]. Finally, the posterior lamina cribrosa of the optic nerve receives perfusion from the peripheral pia arteries and the short posterior ciliary arteries of the Zinn-Haller arterial circle [35].

As mentioned above, there were no differences in the ONH parameters based on sex in the current study; however, long-term follow-ups of both the men and women are necessary to confirm these findings. This is because there are sex-dependent differences in the body’s response mechanisms to SARS-CoV-2 infection. For example, women, after a viral infection, have a different expression of ACE2 and TMPRSS2 receptors, lower production of proinflammatory interleukin-6, and increased activity of innate immune cells against pathogens [31,36,37,38]. Higher levels of estrogen-regulated ACE2 receptors in women play a protective role against lung injury, while the *TMPRSS2* gene is regulated by an androgen-responsive promoter. In addition, estrogen promotes both innate and acquired immune responses, whereas testosterone suppresses immune function and counteracts estrogen-influenced pathways, potentially leading to greater susceptibility to infectious diseases in men [39,40]. Furthermore, female hormones contribute to a protective effect on ocular vascularization when vascular resistance in the large vessels of the eye is reduced [41,42].

We observed positive correlations between the RPC and RNFL optic disc parameters in the four ONH quadrants both in the COVID-19 patients and the healthy controls. Similarly, Savastano et al. [29] found a linear relationship between the peripapillary RNFL thickness and RPC plexus perfusion density and the RPC plexus flow index in patients from the COVID-19 group. The deterioration of blood supply to the optic nerve may cause the thinning of the peripapillary RNFL [29].

The regulation of blood flow through the retina and optic nerve and the maintenance of an adequate oxygen and nutrient supply to these structures are independent of any fluctuations in metabolic demand, pressure driving the blood flow, or changes in the oxygen and carbon dioxide content. This is necessary for the proper functioning of the retina and optic nerve. The regulation of blood flow in these areas involves systemic control (activation of the sympathetic nervous system) and local control (modification of the smooth muscle tone) [43]. However, local factors, such as nitric oxide, prostaglandins, endothelin, and the renin-angiotensin system, play a more significant role in regulating circulation than a systemic control [44,45]. Additionally, the autoregulation of blood flow in the ONH is extensive, similar to that in the retina [46]. The fluctuations in the arterial blood oxygen content observed in SARS-CoV-2 infection promote reversible changes in ONH perfusion. The elevated concentrations of carbon dioxide in the arterial blood can increase the blood flow in the ONH as well as in the retinal and cerebral circulation [47].

In our opinion, the lack of differences in the RPC parameters between the COVID-19 patients and the healthy controls in our study may be due to the good perfusion of the optic nerve. In our previous work, we found an increase in the ganglion cell layer and RNFL thickness in several areas of the central retina in patients after SARS-CoV-2 infection [12]. These changes may be attributed to the vascularization of the inner retinal layers. The ganglion cell layer is supplied with blood by the superficial capillary plexus, which is the final branch of the central retinal artery. The ischemia and secondary atrophy of the inner retina in COVID-19 patients may result from endotheliitis and microthrombotic processes within these small vessels. The avascular zone of the macula is supplied with blood by the choroid, which is one of the most vascularized tissues in the human body [12]. Therefore, disturbances in choroidal blood flow may also contribute to retinal changes observed in patients with COVID-19.

This study has several limitations. First, patients were assessed 2 months after hospital discharge and not during hospitalization for COVID-19 bilateral pneumonia. This was due to organizational factors, patient conditions, and the need to protect the research staff from infection. Second, the study population did not include critically ill patients requiring admission to the intensive care unit or those with progressive hypoxia. Such patients often show an increased release of proinflammatory and prothrombotic factors that could affect the ONH microvascular parameters. Third, a larger study group could have provided more information on the impact of SARS-CoV-2 infection on ONH microcirculation. The strengths of this study include the young age of patients and the absence of additional diseases, especially vascular ones because it would not be possible to rule out the influence of such diseases on the RPC parameters. This increases the reliability of our results and allows the assessment of how such factors as inflammation, hypoxia, and hypercoagulability affect RPC parameters.

## 5. Conclusions

In our study, we found no differences in the RPC parameters between the COVID-19 patients and the healthy controls. In contrast, we found a positive correlation between the RNFL optic disc and RPC parameters. Additionally, no sex-related differences in the RPC parameters were identified among the patients who recovered from SARS-CoV-2 infection. We can conclude that both local and systemic factors effectively regulate the steady blood flow through the retina and optic nerve, resulting in no differences in the RPC parameters between the COVID-19 patients and the healthy controls. Furthermore, the blood flow through these structures is sufficiently preserved even in the case of fluctuations in blood oxygen and carbon dioxide content. Nevertheless, the OCT findings in patients who recovered from COVID-19 should be carefully monitored, especially in those with severe disease.

## Figures and Tables

**Table 1:** Demographic, ocular, and systemic characteristics of the COVID-19 and control groups.

Variable	COVID-19 Group	Control Group	*p*-Value
Men, n (%)	43 (68.25)	28 (62.22)	0.515
Women, n (%)	20 (31.75)	17 (37.78)	
Age, years, mean (SEM); median (IQR)	51.33 (1.45); 51.00 (18.00)	47.76 (1.38); 47.00 (10.00)	0.087 *
BMI, kg/m^2^, mean (SEM), median (IQR)	28.41 (0.51); 28.00 (6.00)	26.77 (0.64); 26.50 (6.00)	0.047 *
LogMar visual acuity, mean; median (IQR)	0.0; 0.00	0.0; 0.0	–
LogMar reading vision, mean; median (IQR)	0.3; 0.03	0.3; 0.0	–
Spherical equivalent, D, mean (SEM); median (IQR)	0.13 (0.13); 0.0 (2.25)	−0.67 (0.13); 0.63 (1.37)	<0.001
Axial length, mm, mean (SEM); median (IQR)	23.55 (0.08); 23.45 (1.05)	23.35 (0.10); 23.45 (1.22)	0.111 *
Intraocular pressure, mmHg; mean (SEM); median	16.16 (0.24); 15.90	16.36 (0.34); 16.40	0.623
Hypertension, n (%)	19 (30.16)	–	–
Dyslipidemia, n (%)	3 (4.76)	–	–
Oxygen therapy, n (%)	22 (34.92)	–	–

* Student *t*-test. Abbreviations: BMI, body mass index; D, diopter; IQR, interquartile range; SEM, standard error of the mean.

**Table 2:** Comparison of the optic nerve head parameters between the COVID-19 and control groups.

Variable	COVID-19 Group		Control Group		*p*-Value
	Mean (SEM)	Median (IQR)	Mean (SEM)	Median (IQR)	
Rim area (mm^2^)	1.45 (0.03)	1.39 (0.53)	1.45 (0.04)	1.43 (0.43)	0.772 *
Disc area (mm^2^)	1.98 (0.03)	1.98 (0.39)	1.91 (0.04)	1.87 (0.39)	0.150 *
Linear CDR	0.47 (0.02)	0.49 (0.25)	0.44 (0.02)	0.49 (0.21)	0.403 *
Vertical CDR	0.46 (0.02)	0.48 (0.27)	0.44 (0.02)	0.50 (0.22)	0.547 *
Cup volume	0.11 (0.01)	0.06 (0.14)	0.07 (0.01)	0.05 (0.08)	0.353 *

* Mann-Whitney test. Abbreviations: CDR, cup-disc ratio; IQR, interquartile range; SEM, standard error of the mean.

**Table 3:** Comparison of the radial peripapillary capillary parameters between the COVID-19 and control groups.

Variable	COVID-19 Group		Control Group		Effect Size	*p*-Value
	Mean (SEM)	Median (IQR)	Mean (SEM)	Median (IQR)		
RPC S	51.47 (0.25)	51.29 (3.65)	50.97 (0.36)	51.56 (3.23)	0.06	0.432 *
RPC N	44.68 (0.35)	45.39 (4.27)	44.89 (0.51)	45.58 (4.85)	–0.02	0.754 *
RPC I	52.48 (0.25)	52.40 (3.20)	52.31 (0.35)	52.40 (3.22)	0.01	0.943 *
RPC T	45.79 (0.24)	45.88 (3.24)	45.84 (0.34)	46.19 (2.91)	–0.04	0.559 *
Mean RPC	48.60 (0.15)	48.77 (2.29)	48.50 (0.27)	49.02 (2.63)	–0.03	0.669 *

* Mann-Whitney test. Abbreviations: I, inferior; IQR, interquartile range; N, nasal; RPC, radial peripapillary capillary; S, superior; SEM, standard error of the mean; T, temporal.

**Table 4:** Comparison of the radial peripapillary capillary parameters between the men and women in the COVID-19 group.

Variables	Men		Women		Effect Size	*p*-Value
	Mean (SEM)	Median (IQR)	Mean (SEM)	Median (IQR)		
RPC S	51.19 (0.31)	51.14 (4.04)	52.08 (0.41)	52.60 (3.67)	–0.15	0.107 *
RPC N	44.55 (0.40)	45.27 (4.24)	44.96 (0.70)	45.49 (4.38)	–0.10	0.303 **
RPC I	52.46 (0.28)	52.28 (0.28)	52.53 (0.49)	52.50 (4.12)	–0.04	0.709 **
RPC T	45.97 (0.27)	45.99 (3.29)	45.38 (0.49)	45.37 (3.8)	0.11	0.266 *
Mean RPC	48.54 (0.16)	48.75 (2.3)	48.74 (0.33)	48.91 (2.77)	–0.06	0.559 *

* Student *t*-test, ** Mann-Whitney test. Abbreviations: I, inferior; IQR, interquartile range; N, nasal; RPC, peripapillary vessel density; S, superior; SEM, standard error of the mean; T, temporal.

**Table 5:** Correlations between the radial peripapillary capillary and retinal nerve fiber layer optic disc parameters in the COVID-19 group.

Variable	R_Sp_	*p*-Value
RPC S vs. RNFL optic disc S	0.54	<0.001
RPC N vs. RNFL optic disc N	0.63	<0.001
RPC I vs. RNFL optic disc I	0.55	<0.001
RPC T vs. RNFL optic disc T	0.52	<0.001
Mean RPC vs. mean RNFL optic disc	0.51	<0.001

Abbreviations: I, inferior; N, nasal; RCP, radial peripapillary capillary; R_Sp_, Spearman rank correlation coefficient; S, superior; T, temporal.

**Table 6:** Correlations between the radial peripapillary capillary and retinal nerve fiber layer optic disc parameters in the control group.

Variable	R_Sp_	*p*-Value
RPC S vs. RNFL optic disc S	0.59	<0.001
RPC N vs. RNFL optic disc N	0.73	<0.001
RPC I vs. RNFL optic disc I	0.64	<0.001
RPC T vs. RNFL optic disc T	0.64	<0.001
Mean RNFL vs. mean RCP	0.69	<0.001

Abbreviations: I, inferior; N, nasal; RCP, peripapillary vessel density; R_Sp_, Spearman rank correlation coefficient; S, superior; T, temporal.

## Data Availability

The data supporting the reported results can be provided upon request from the corresponding author.
